# Thermal Boost to Breast Tumor Bed—New Technique Description, Treatment Application and Example Clinical Results

**DOI:** 10.3390/life12040512

**Published:** 2022-03-30

**Authors:** Adam Chicheł, Wojciech Burchardt, Artur J. Chyrek, Grzegorz Bielęda, Grzegorz Zwierzchowski, Patrycja Stefaniak, Julian Malicki

**Affiliations:** 1 Department of Brachytherapy, Greater Poland Cancer Center, 61-866 Poznan, Poland; wojciech.burchardt@wco.pl (W.B.); artur.chyrek@wco.pl (A.J.C.); 2 Department of Electroradiology, Poznan University of Medical Sciences, 61-866 Poznan, Poland; grzegorz.bieleda@wco.pl (G.B.); grzegorz.zwierzchowski@wco.pl (G.Z.); julian.malicki@wco.pl (J.M.); 3 Department of Medical Physics, Greater Poland Cancer Center, 61-866 Poznan, Poland; patrycja.stefaniak@wco.pl

**Keywords:** thermal boost, hyperthermia, breast cancer, interstitial brachytherapy, treatment planning

## Abstract

(1) Current breast-conserving therapy for breast cancer consists of a combination of many consecutive treatment modalities. The most crucial goal of postoperative treatment is to eradicate potentially relapse-forming residual cancerous cells within the tumor bed. To achieve this, the HDR brachytherapy boost standardly added to external beam radiotherapy was enhanced with an initial thermal boost. This study presents an original thermal boost technique developed in the clinic. (2) A detailed point-by-point description of thermal boost application is presented. Data on proper patient selection, microwave thermal boost planning, and interstitial hyperthermia treatment delivery are supported by relevant figures and schemes. (3) Out of 1134 breast cancer patients who were administered HDR brachytherapy boost in the tumor bed, 262 were also pre-heated interstitially without unexpected complications. The results are supported by two example cases of hyperthermia planning and delivery. (4) Additional breast cancer interstitial thermal boost preceding HDR brachytherapy boost as a part of combined treatment in a unique postoperative setting was feasible, well-tolerated, completed in a reasonable amount of time, and reproducible. A commercially available interstitial hyperthermia system fit and worked well with standard interstitial brachytherapy equipment.

## 1. Introduction

The current standard of radical treatment for most breast cancer (BC) patients is breast-conserving therapy (BCT). It consists of conservative surgery, radiotherapy ± brachytherapy (BT), optional adjuvant chemotherapy, and hormonal therapy if indicated. In early invasive BC treatment, BCT is a good alternative to mastectomy [1,2,3,4,5]. The goal of administering irradiation is to minimize the local relapse risk in the treated breast. Thus, the area of the most interest is the tumor bed, which is the target volume for BT. Worldwide, many solutions exist for a local dose increase (boost) in the tumor bed volume [6,7]. Contemporary available interstitial multi-catheter (MC) high-dose-rate (HDR) BT has the potential for the most conformal and precise target volume irradiation. Preferably, it should be proposed especially to young patients aged < 50 years with a close, microscopically positive, or unknown surgical margin and with negative factors that increase the probability of local relapse [8,9,10]. The boost may reduce the five-year local recurrence rate from 7.3–13.3% to 3.6–6.3% (mean 5.5%) [9,11,12,13].

There is a margin for further improvement; thus, finding a regimen enabling up to 100% local control (LC) would be valuable. That goal can be obtained by eradicating the complete gross tumor and all cancer cells potentially left in the surrounding healthy breast tissue. Additional local hyperthermia (HT), which has already proved its cancer treatment augmentation potential, is one of the therapeutic options [14,15].

In the clinic, HT enables an artificial increase in the temperature of areas of the human body, especially those containing cancerous tumors [16,17]. Heat must be delivered by external sources, e.g., microwaves (MW), to increase the temperature in the region of interest up to 40–43 °C, and this elevated temperature is maintained for at least 40–60 min [18,19,20]. It has been proved that HT kills cancerous cells and sensitizes them to radiation (among others), usually with no harm to normal tissues [18,20,21,22,23], and is often used in conjunction with other therapies [19,24]. The most oncologically effective approach is delivering HT simultaneously with radiation, which is almost impossible in clinical practice. However, tight time constraints can be met, especially in a brachytherapy suite environment.

In 2006, we started adding a single preceding HT session (thermal boost, TB) to standard interstitial MC HDR-BT to increase local cure probability. The combination is feasible, as interstitial MW antennas are well suited to standardly used plastic tubes dedicated to interstitial BT. Short- and long-term results regarding the treatment efficacy and toxicity of this combined treatment were previously published [25,26].

Hyperthermia treatment combined with radiation (including interstitial brachytherapy) is an accepted method and is reimbursed in this setting in the authors’ country. Nevertheless, each subject selected and proposed for the thermal boost to achieve an HDR-BT boost provided additional written informed consent.

The main aim of this work is to present a detailed description of how to perform a technique for TB delivery to the breast cancer tumor bed. Based on the authors’ clinical experience, the method seems feasible, safe, completed in a reasonable amount of time, and reproducible.

## 2. Materials and Methods

Between Feb 2006 and Dec 2018, a total number of 1134 stage IA–IIIA breast cancer patients were treated with BCT ± neo- or adjuvant chemotherapy followed by 40.05–50.0 Gy in 2.0–2.67 Gy fractions of whole-breast irradiation (WBI) and 10 Gy single fraction of HDR-BT boost. BT was administered with an ^191^Ir radioactive source (microSelectron-HDR, Nucletron BV/Elekta, Veenendaal, The Netherlands). Of the treated patients, 872 patients (group A; 76.9%) had standard treatment, and 262 patients (group B; 23.1%) were treated with an additional pre-BT single session of interstitial MW HT applied to the tumor bed.

BT was performed with CT-compatible commercially available interstitial applicators, compatible with an OncentraBrachy (Elekta) treatment planning system (TPS). Contemporary proper constraints appropriate for interstitial HDR-BT TPS are suitable for clinical adaptation of the interstitial HT system. The multi-catheter (flexible plastic tubes) implantation technique and treatment planning are described in detail elsewhere [27]. A CT-based planning system requires obtaining three-dimensional (3D) information on in-tissue applicators’ orientations. Of note, rigid metal needles are not suitable for microwaves. MW HT was delivered via a BSD-500 system operating at 915 MHz (BSD Medical Corporation, Salt Lake City, UT, USA). Based on post-implant CT scans, appropriate applicators were selected for insertion with a set of MW antennas and thermal probes to adequately cover the tumor bed within the anticipated range of temperature. The reference temperature (T_ref_) was set at 43 °C. Therapeutic time (TT), corresponding to the time at which the temperature is kept above 40 °C (called therapeutic temperature, T_ter_), was to be maintained for 1 h. Total thermal dose (TTD) was measured for each sensor. The time interval between the HT session cessation and the start of irradiation was kept as short as possible and should not exceed two hours. All activities were performed in accordance with the rules of thermometry and necessary quality assurance (QA) requirements of hyperthermia that have been in force since the release of RTOG guidelines, with subsequent updates [28,29].

Summarizing the above, HDR-BT-related hyperthermia planning and delivery need a trained team of specialists: at least one clinician (radiation oncologist, brachytherapist), medical physicist, radiation therapy technician, and a nurse.

### 2.1. Patient Selection

Patient selection was not randomized and was based on several important individual clinical presentations. Each patient expressed personal agreement with HDR-BT augmentation with MW HT and provided written informed consent. The final decision to add HT was made after implanting the breast and obtaining CT scans. Table 1 summarizes the most critical features needing to be assessed before the determination: adequate breast size, presence of a large and deep-seated tumor bed, skin-to-skin distance, number of application planes, distance to bone structures, and presence of the seroma or hematoma. For each antenna, the mandatory minimum in-breast applicator length is 7 cm to preserve a minimum of 1 cm distance from the antenna’s active length (which is about 5 cm) tip and end to the skin. Maintaining the correct distance from the tumor bed to the skin is vital in preventing skin overheating (experience derived from H&N patients heated interstitially). Fulfillment of this condition prevents burns, pain, and consecutive ulcer formation. MW heating near the bone structures (ribs) and retained fluids (seroma, hematoma) is contraindicated due to their unpredictable and rapid overheating and the lack of natural cooling mechanisms.

### 2.2. MW Thermal Boost Planning

Performing a complete additional thermal boost before HDR-BT may be time-consuming. However, thermal boost planning adds about 10–20 min to the stage of BT treatment planning, depending on the target volume and the number of engaged applicators.

**Figure 1:**
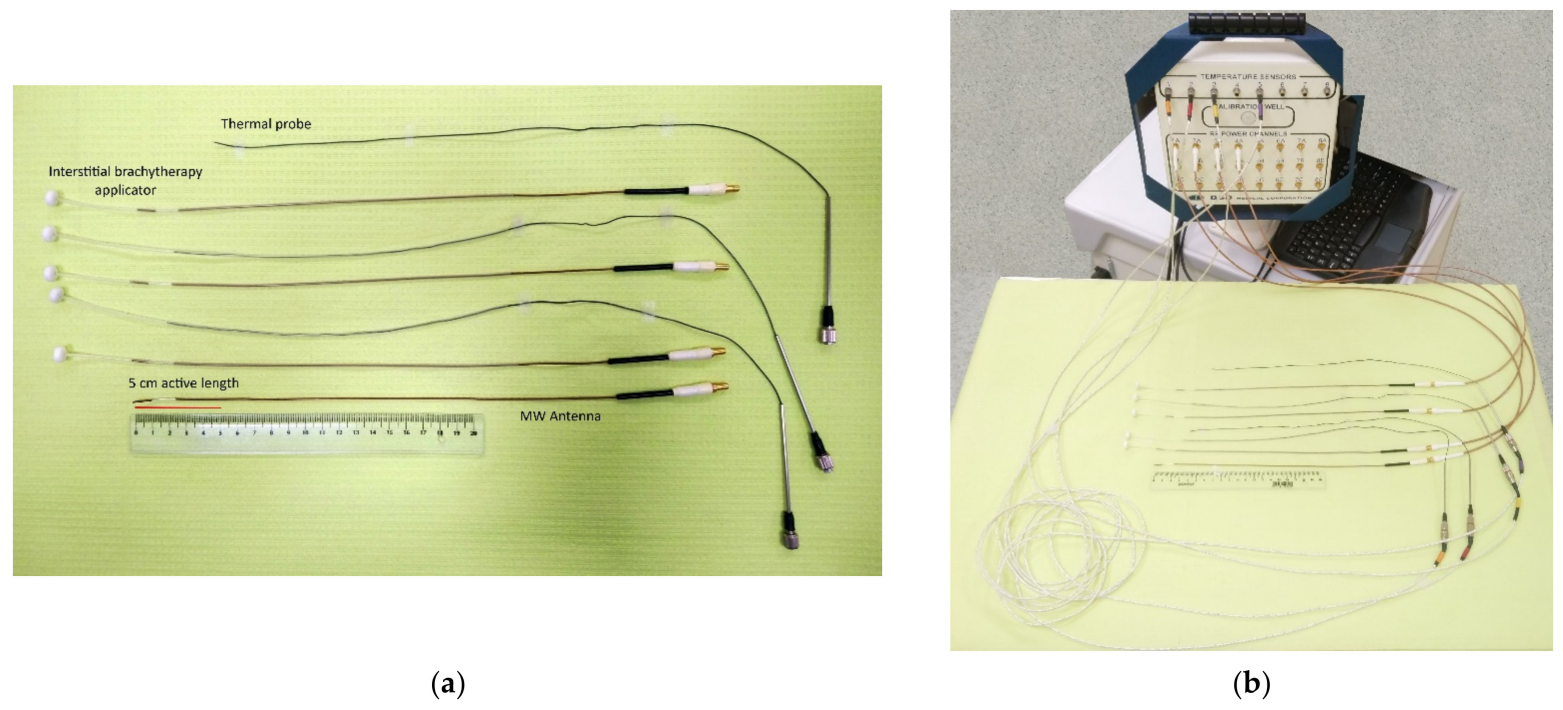
A set of interstitial applicators, MW antennas, and thermal probes (**a**) connected through dedicated wires with the BSD-500 device (**b**).

Figure 1a shows a standard set of five shortened typical interstitial BT applicators, three inserted with interstitial MW antennas and two inserted with thermal probes. A single thermal probe is also presented. Attention should be paid to the single MW antenna with a neighboring ruler that indicates the 5 cm long segment of the active length of the antenna. It also contains eight temperature sensors and a calibration well (Figure 1b).

The BSD-500 system is a compact mobile unit, completely computerized for collecting, displaying, and recording thermometry data. It is equipped with a microwave generator with 32 RF power channels. Each channel can propagate power of up to 50 W. Forward and reflected power can be monitored on each. The clinician or computer, respectively, can manually or automatically control the power balance of up to eight channels. For the interstitial type of heating, MA-250-type MW interstitial antennas are chosen. These thin (1.1 mm diameter) coaxial antennas apply most of their energy from the applicator’s tip. At the time, several should be inserted parallel to each other with a 1–2 cm spacing. For temperature control, it is equipped with a set of temperature sensors (thermometers, Bowman probes, thermal probes) with sensor accuracy of ±0.1 °C. These are thermistor-type sensors with nonmetallic leads, do not perturb MW fields, and are not perturbed by MW fields. A built-in self-controlled thermal well is used for calibration (Figure 1a,b).

**Figure 2:**
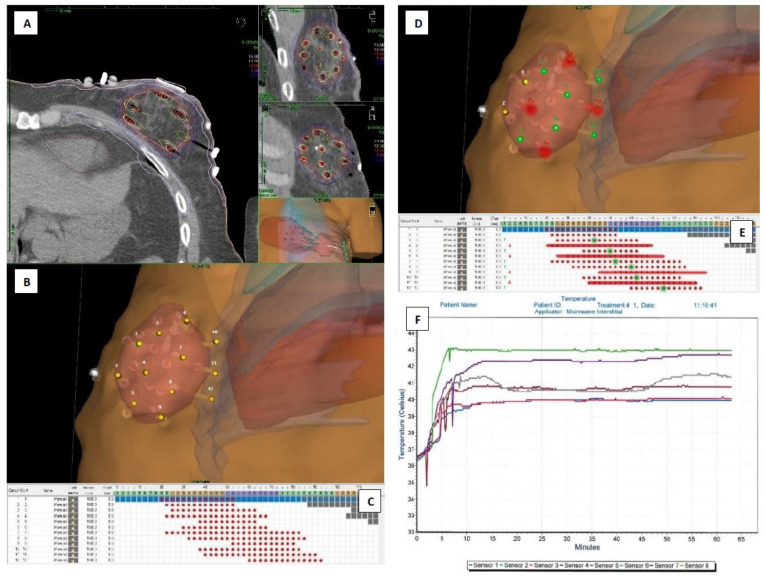
Case 1 planning and temperature measurement results—presentation: (**A**) cross-, sagittal- and frontal CT plane sections with optimized isodoses display, (**B**) applicator axis adjusted 3D view of the interstitial implant protruding the delineated tumor bed—salmon color, (**C**) source positions activated inside consecutive applicators—red dots, (**D**) applicators selected for 4 MW antennas (red) and 6 thermometers (green) insertion, (**E**) antennas active lengths adjusted to source positions—red lines, planned optimal positions for thermometers’ tips—green dots, (**F**) graphical presentation of the whole HT session temperature measurements with reference thermometer in green.

**Figure 3:**
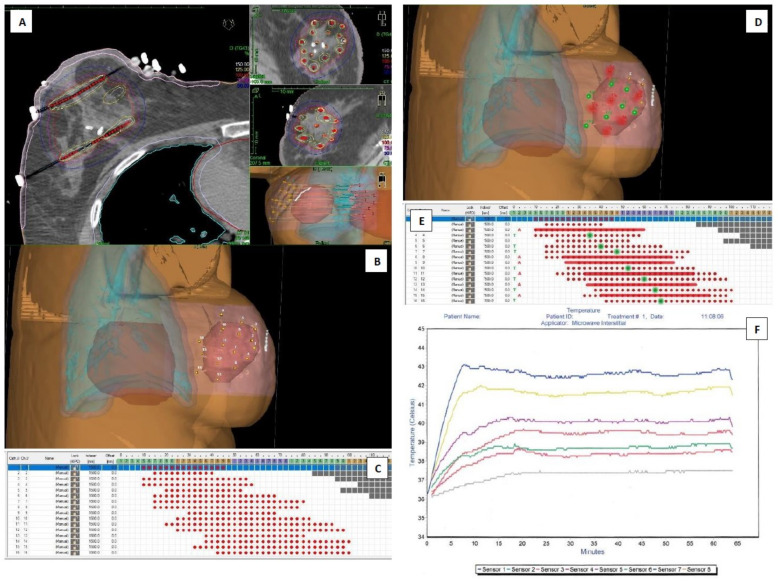
Case 2 planning and temperature measurement results—presentation: (**A**) cross-, sagittal- and frontal CT plane sections with optimized isodoses display, (**B**) applicator axis adjusted 3D view of the interstitial implant protruding the delineated tumor bed—salmon color, (**C**) source positions activated inside consecutive applicators—red dots, (**D**) applicators selected for 6 MW antennas (red) and 7 thermometers (green) insertion, (**E**) antennas active lengths adjusted to source positions—red lines, planned optimal positions for thermometers’ tips—green dots, (**F**) graphical presentation of the whole HT session temperature measurements with reference thermometer in blue.

**Scheme 1:**
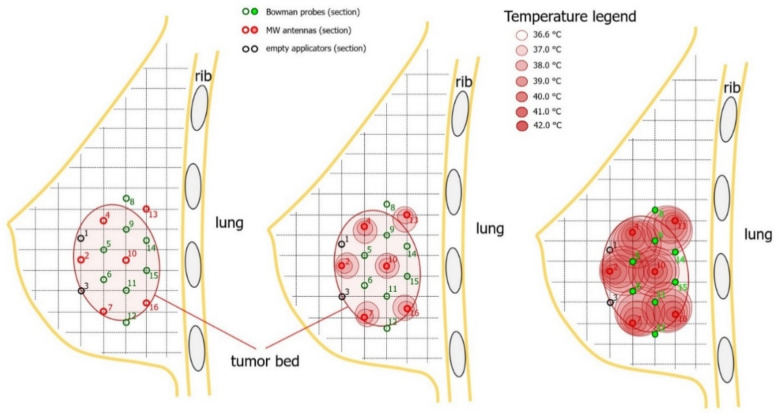
Schematic sagittal breast presentation with delineated tumor bed, cross-sectional view of the four-plane interstitial application, and proposed antennas and Bowman probes.

**Scheme 2:**
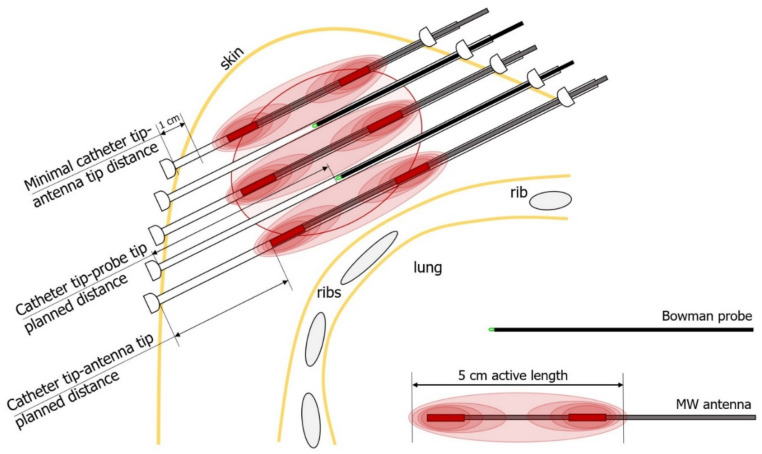
Schematic breast cross-section presentation of a tumor bed, sagittal view of the five-plane interstitial application, and proposed placement of antennas and Bowman probes. Significant minimal distances to the skin and the antenna’s active length are presented.

Point-by-point MW HT planning:

1. Regular interstitial application is performed according to the GEC-ESTRO recommendations [30], with local institutional adjustments regarding accessible equipment (pre-BCT mammography and USG, complete post-surgical pathological report, EBRT planning CT, clinical examination, C-arm X-ray in the operating room, intraoperative USG).
2. CT scanning with ≤3 mm thick layers.
3. Regular 3D initial brachytherapy plan preparation in the TPS: structure reconstruction (patient body, lungs, heart, skin, chest wall, ribs, surgical clips, skin scar) and CTV—visible tumor bed (postoperative scarification) plus adequate safety margin according to pathological report and recommendations, as well as applicator reconstruction (Figure 2A,B and Figure 3A,B).
4. The most critical stage is selection of the proper applicator to be inserted with MW antennas and thermometers (Scheme 1 and Scheme 2):
  − Three-dimensional presentation of the breast and interstitial application need to be inspected and assessed to determine if thermal boost is possible in a particular case;
  − One needs to answer the question of whether the applicators are implanted in a regular, equidistant, and parallel manner and whether the insertion of 4–6 antennas is possible while leaving free applicators for temperature measurement between antennas;
  − It must be remembered that the temperature needs to be monitored, especially in the tumor bed volumes expected to be heated most; the best view for this is a section perpendicular to the application Figure 2B and Figure 3B; Scheme 1, left);
  − One must make sure that the expected antenna configuration fits the tumor bed delineated as CTV in the TPS; this is the stage when thermal boost volume is adjusted to the brachytherapy boost volume, and it is only possible when based on a clinical inspection by the planning physician;
  − Referring to the scheme (Scheme 1, middle and right; Figure 2D and Figure 3D), applicators for antennas are selected to best cover the CTV, and then applicators for thermal probes are chosen to ensure relevant temperature monitoring, especially in spots anticipated to be the hottest (e.g., Scheme 1, right, applicator number 5);
  − Then, longitudinal orientation has to be inspected to determine if other requirements can all be met (Scheme 2, Figure 2A and Figure 3A): distances from each antenna’s active length to the skin and ribs.
5. Next, it is mandatory to define the exact positions of each antenna and thermometer in a dedicated applicator. For that purpose, in the TPS, the physicist activates source positions in each applicator protruding from the CTV (Figure 2C and Figure 3C):
  − Having chosen the antenna-dedicated applicators (Figure 2E and Figure 3E; red capital letter A), the planning physician decides on the antenna position in the applicator lumen, which should be the place that ensures that the CTV is covered by the antenna’s 5 cm active length;
  − Then, the distance from the applicator tip end to the tip end of the antenna has to be measured and noted for further reference; the same process is performed for all antennas;
  − Having chosen the thermometer-dedicated applicators (Figure 2E and Figure 3E, green capital letter T), the planners decide on the thermal probe (sensor) tip position in the applicator lumen. The probe tip should be positioned in the middle of neighboring antennas’ active lengths, where the highest temperature is expected to be measured. In addition, according to recommendations [28,29], planners should provide sensors on the periphery (e.g., Scheme 1, right, applicator numbers 8, 12, 14, 15). The BSD-500 provides up to 8 sensors. Due to the limited number, their positions should be effectively chosen with care.
  − Then, the distance from the applicator tip end to the tip end of the probe has to be measured and noted for further reference; the same process is performed for all thermal probes.
6. The HT session plan is ready.

In the meantime, after being scanned, the patient is monitored by the nurse and awaits the start of treatment in a comfortable lying position. If necessary, she is administered painkillers.

### 2.3. MW Thermal Boost Delivery

Each HT session was planned to achieve a TT of 60 min. Ultimately, it took about 80–90 min, including the preparation and insertion of antennas and thermometers into the tubes. The physicist ± physician may perform this step; however, a well-trained RTT is the most suitable and competent for this task.

Before the start of treatment, the patient is to be instructed on the feelings that she may experience. The tips are comfortably and precisely transferred to the patient when the antennas and sensors are inserted into the applicators. This process is carried out precisely according to the noted distances measured for dedicated tubes. Antennas are manually taped to the applicators’ external tips to prevent movement. Thermometers are also positioned and partially taped, but one to three are left free for thermal mapping.

The operator uses a touch-screen monitor to control all necessary parameters: selects the number of thermal probes and reads the current temperature captured by the sensors, chooses the number of antennas to be used, and sets each antenna’s output power. All sensors intended to be used are calibrated in the calibration well before the HT session starts. When the above preparations are complete, the power is turned on, and MW energy starts to be propagated through antennas to the tissue. The probes start reporting increasing temperature, displayed as temperature graphs on the monitor (Figure 2F and Figure 3F).

As part of QA, thermal mapping is possible and recommended for each channel separately. With experience gained in other clinical settings, pretreatment decisions on the most probable reference thermometer (TM_ref_) location save time. It should be placed in the sub-volume of a heated target located between the most closely spaced antennas, where the fastest T_ter_ and T_ref_ measurements can be made. The most reasonable approach is to start thermal mapping with the assumed TM_ref_. Then, the operator records temperatures from a selected sensor that is manually positioned along a catheter track. Once the highest temperature reading is found (Figure 2F; e.g., sensors: 6 green, 5 purple, 4 brown), the sensor is taped to the catheter to secure its position. The sensor measuring the highest temperatures is set as TM_ref_. The temperature control system is used to limit RF power based upon the T_ref_ maximum temperature level setting, which is 43 °C in the present case. If achieved, the system automatically controls the RF power to reduce the heating and ensure delivery of a power level that can sustain the desired temperature and prevent exceeding the set limit. It is important to monitor other sensors’ readings and react accordingly if the temperature reaches the T_ref_ in another target subvolume.

The system starts recording TT from the moment that T_ter_ is achieved in any sensor. If adequately chosen during planning, it most often occurs first in the assumed TM_ref_ (Figure 2B,D,F: sensor 6 (green) in catheter 7; Figure 3B,D,F: sensor 1 (blue) in catheter 12). The system switches off the power once the set 60 min TT elapses. The cooling process then starts. Two to three minutes of temperature monitoring is advised to ensure proper cooling of the heated volume. Then, it is time to disassemble the antennas and sensors and prepare the patient for HDR-BT treatment as soon as reasonably possible.

From the patient’s point of view, a feeling of increasing non-impairing warmth inside the implanted breast is normal. This feeling increases until the reference temperature is achieved, after which the feeling diminishes. Automatic power adjustments cause this effect. Then, the power is lowered and oscillates between lower values than initially, so the patient feels less heating. In rare cases, the patient may complain of local, point-like sharp skin pain. In that case, the antenna may be too close to the skin and needs to be moved forward or backward (depending on the side of pain occurrence), or in the single channel, propagated power has to be decreased to release the pain. Each antenna adjustment necessitates another thermal mapping with the nearest thermal probe. Whenever these actions fail to solve the problem, the clinician has to decide whether to switch off the problematic antenna or, if necessary, stop the HT session altogether.

### 2.4. HDR Brachytherapy Boost Delivery

Usually, when the workflow is well organized, the patient is ready to start BT irradiation 20 to 30 min after the HT session is finished. If the brachytherapy suite is equipped with the HT system, the important requirement of combining the methods within 2 h is easily feasible. After the irradiation is completed, the interstitial applicator is removed from the breast tissue, followed by necessary compression and final dressing application.

The whole procedure often takes about 4 to 5 h (TB + HDR-BT), and the patient is discharged home on the same day.

## 3. Results

All 262 patients who started TB completed the heating and received BT irradiation without unexpected complications. The authors did not report any technical problems regarding the flexible plastic tubes used for heating. The time from HT onset to the therapeutic time start point typically took 2–3 min. The time from HT onset to the maximum value of T_ref_ took 6–8 min. The median number of antennas used for each session was 4 (range 3–6). The median number of interstitial thermometers was 4 (range 3–7). The mean TM_ref_ measurements were 41.6 °C, and the median was 42.2 °C (39.2–42.8 °C). Median TT (≥40 °C) was 61.4 min (range 0–65.3 min). The median gap between HT sessions and HDR-BT delivery was 30 min (range 5–60 min), so all patients received the proper, timely combination of HT with irradiation criteria. The results were very similar to previously published data on 32 HT cases [25].

We present two example cases (Figure 2 and Figure 3A–F) to track the planning and temperature monitoring process. The figures contain a series of logically arranged screenshots from the TPS and HT system temperature report. They are referred to and discussed in detail in the Materials and Methods section.

**Figure 4:**
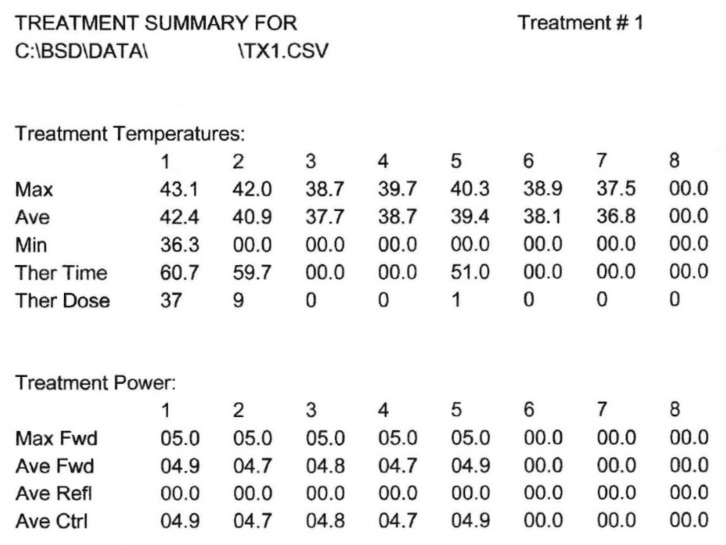
HT session report, corresponding to the case presented in Figure 3. Abbreviations: Ther Time—therapeutic time, TM_ter_; Ther Dose—therapeutic dose; Max/Ave Fwd—maximum/average energy propagated forward the MW antenna; Ave Refl—average power reflected by MW antenna.

Figure 4 shows an example of a heating session report derived from the system. The data graphically correspond to the temperature graph presented in Figure 3F. As illustrated, three sensors (1, 2, and 5) exceeded T_ter_. Only TM_ref_ (sensor 1) achieved T_ref_ and controlled the power output during the session. In the example, the average power for working antennas ranged from 4.7 to 4.9 W. Overall, the mean MW energy for particular HT sessions was 3.7 W (range 1.4–5.7).

Reported patient complaints were scarce. At the start of the HT session, if a patient reported heat-induced point pain, the above-proposed adjustments satisfactorily solved the problems. No sessions were terminated for that reason. There was no premature discontinuation of treatment, as patients were properly selected in all cases.

The rest of the long-term clinical results are being analyzed and prepared for final data presentation in an anticipated publication.

Early and late side effects in the group of initial patients were assessed earlier in other series [25,26]. Compared to sole HDR-BT, thermal boosting did not increase any bleeding problems (neither profuse nor prolonged) after irradiation completion and applicator removal. Any breast enlargement or edema due to heating was noted, and there was no need for HDR-BT treatment plan adjustments or re-planning.

## 4. Discussion

The present paper describes additional breast cancer thermal boost preparation and delivery as a part of combined treatment in a unique postoperative setting. Most of the available data concerning HT in breast cancer treatment relate to inoperable, locally advanced, or recurrent disease. To our knowledge, no other attempts have been undertaken to thermally boost postoperative breast irradiation as a breast-conserving approach with either an interstitial or external method.

Dooley et al. investigated pre-mastectomy-focused MW thermotherapy of early-stage BC and suggested a reduction in the incidence of positive margins (0 vs. 9.8%) in heated patients [23]. Postoperative EBRT and HDR-BT boost aim to eradicate potentially invasive cells left by the surgeon in the tumor bed surrounding. HT aims to sensitize them to radiation, thus enhancing the treatment efficacy.

In 1997, Hartmann et al. [31] published their results on 158 preoperative BC treatments combined with interstitial HT and, similarly to ours, 10 Gy interstitial BT boost immediately preceded a single local HT session. However, they did not provide instructions on carrying out the HT session. Ryan et al. [32], in their summary on contemporary interstitial MW treatment for cancer, included some critical data on antenna arrays in mild thermal treatment: thermal dose objectives, image guidance, placement, temperature measurements, treatment planning, and real-time assessment, although they focused on the transition of interstitial HT to thermal ablative treatment.

In 2020, Kok et al. [33] published a review on current heating technologies for malignant tumors. Worldwide, local interstitial MW HT is utilized as a radiosensitizer in a few locations: breast, head and neck, and prostate cancers, and with tumors in situ. The QA guidelines for interstitial HT by Dobšíček Trefná et al. [29] underline the distribution, alignment, and catheter spacing determined by the BT constraints and requirements. Because irradiation is a crucial part of treatment, implants may be suboptimal for high-quality HT delivery. Interstitial application imperfections may be overcome by the power control of individual antennas with accurate thermal mapping. However, the operator must be aware that heterogeneous catheter spacing can result in hot and cold spots in the implant, and one might not identify some of them. Again, the guidelines relate to heating the tumor, not the tumor bed. Nevertheless, even though the technique presented here was developed in 2006, since the very beginning, it has been firmly based on recommendations [28]. The step-by-step instructions given above in the MW HT planning and delivery sections reflect current guidelines.

The most similar clinical scenario to that in the study is the one presented by van Vulpen et al. [34] and Kukiełka et al. [35]. In their works, interstitial HT was added to interstitial BT in the treatment of prostate cancer (PCa). The similarity is that the prostatic tumor was heated and irradiated, and, of note, the remaining healthy tissue of the gland was as well. Treated volumes were adjusted to each other, and the same interstitial applicators were used for both modalities. In Kukiełka’s studies, HT was added to enhance the therapeutic ratio, with no apparent increase in toxicity. They investigated several clinical presentations: HT combined with HDR monotherapy, HT combined with HDR-BT boost after EBRT, and HT combined with salvage HDR-BT for recurrent PCa. They performed HT using the same BSD-500 system and the same recommendations for heating planning and delivery [35,36,37]. They also developed a schematic approach to HT session planning with established average positions for HT antennas and thermal probes [35].

In contrast to their scheme, we measure temperature in each tissue portion suspected of being heated most, ensuring the lack of overheated spots. Next, they reported no excess bleeding after interstitial applicator removal once the treatment was completed, and we observed the same. In slight contrast to our experience, the additional time prolonged the standard HDR-BT procedure by as little as 15 to 30 min. The interval between HT completion and irradiation onset lasted only 5 to 15 min. Kudzia et al. [38] investigated the potential HT influence on the prostatic volume. They found no significant changes in volume and treatment plan parameters due to heating. This is in concordance with our observations, although it was impossible to make such image-based measurements. It is also worth noting that our patients are entirely conscious, not anesthetized, and give feedback on any pain or concerning feelings during the HT session.

Regarding breast cancer, external systems are often used [39]. Arunachalam et al. [40] designed ThermoBrachytherapy Surface Applicator (TBSA) for combined and almost simultaneous thermobrachytherapy of chest wall recurrences. They reported a thermal enhancement ratio as high as 2.5. Some researchers investigated external systems of focused MW phased array thermotherapy for primary breast cancer in preoperative settings [41,42,43]. These systems should not be compared with the interstitial approach, as these techniques differ substantially.

Nevertheless, the work of Nguyen et al. [44] is worth noting. They presented a comprehensive model of a complete non-invasive HT system in a suitable 3D environment, including a realistic breast model and realistic antennas. The results showed that the HT treatment could be focused on the exact volume of tumors seated in different breast parts while preventing the generation of hot spots in healthy tissue. In the current study and studies discussed earlier [35,36,37] concerning the interstitial approach, the heated volume is adjusted to the clinical target volume with high precision, which is much more manageable.

At the time of method implementation, the authors had no external instructions on how to heat the tumor-free target and developed their original method described above. No other research was found concerning non-strict tumor heating with potentially cancerous cell-containing tissue. Finally, it appears to be feasible and very well tolerated in the clinic. Our quite significant group of patients is now being evaluated, and the results on treatment efficacy and late toxicity are to be presented soon.

## 5. Conclusions

Based on the authors’ clinical experience, additional breast cancer interstitial thermal boost preceding HDR brachytherapy boost as a part of combined treatment in a unique postoperative setting is feasible, well-tolerated, completed in a reasonable amount of time, and reproducible. The commercially available interstitial hyperthermia system fit and worked well with standard interstitial brachytherapy equipment.

## Figures and Tables

**Table 1:** Tumor bed features suitable for safe and efficient interstitial MW HT procedure.

Feature	Suitable	Unsuitable
Skin-to-skin distance	≥7 cm	<7 cm
Applicator tip– antenna tip/end distance	≥1 cm	<1 cm
Number of planes	≥3	<3
Minimum antenna–skin distance	≥1 cm	<1 cm
Antenna–rib(bone) distance	≥1 cm	<1 cm
Seroma/hematoma	absent	present

## Data Availability

The data presented in this study are available on request from the corresponding author. The data are not publicly available due to restrictive personal data protection solutions in force in the authors’ institution.
